# Book Reviews

**Published:** 1896-07

**Authors:** 


### BOOK REVIEWS.

A MANUAL OF ANATOMY. By Irving S. Haynes, M.D., Adjunct and Demonstrator of Anatomy in the Medical Department of the University of the City of New York; Member of the American Association of Anatomists, etc. One hundred and thirty-four half-tone illustrations and forty-two diagrams. Published by W. B. Saunders, 925 Walnut street, Philadelphia. Price, $2.50.

Systematic and exhaustive treatises on human anatomy are by no means wanting, and of compends there is certainly an abundance; but how to strike the happy medium between these two and produce a practical working manual for the student at the dissecting-table seems to be a difficult matter. In this book Dr. Haynes has succeeded about as well as others who have written for the same purpose. Dr. Haynes seems to appreciate the student's needs, and has written for the student. He has eliminated from his descriptions a great deal of unnecessary matter, and has included very little that is not important and essential. The regional method of description which he wisely follows is the only rational method of presenting the subject for dissecting purposes. The half-tone illustrations are from photographs of actual dissections, and they therefore give the student pictures the like of which he may hope to reproduce for himself in his own dissections. Some of these illustrations, however, are made too small to be of much service: *e. g.*, figure 83, figure 102, figure 111, figure 123, figure 67, and figures 129 and 130. But the illustrations, as a rule, are good, much better than the highly colored pictures in some of the books, which are evolved out of the imaginations of artists. The anatomy of the brain occupies more than its proper proportion of space, one hundred pages being devoted to it. Ligaments are omitted altogether, an error which the author should certainly correct in the next edition.

Upon the whole the work is to be commended. The author thoroughly knows his subject, and his book will help to efficiently supply the wants of the dissecting-room.

AN AMERICAN TEXT-BOOK OF SURGERY. For Practitioners and Students. Edited by William W. Keen, M.D., LL.D., and J. William White, M.D., Ph.D. Second edition, carefully revised. Philadelphia. W. B. Saunders, 925 Walnut St., Philadelphia.

The popularity of the "American Text-Book of Surgery" is evinced by the early exhaustion of the first edition, and its adoption as a text-book by many of the medical schools of the country. The second edition has been thoroughly revised, and many new additions have been made to the text; new illustrations have been added, and some of the chapters have been entirely rewritten, bringing the book well up to date. This is especially true of the sections dealing with fractures, dislocations, appendicitis, radical cure of hernia, and amputations of the breast. The work as a whole is a most excellent one, and abundantly merits its almost universal popularity. Among the contributors are Drs. Frederic S. Dennis, Roswell Park, L. S. Pitcher, Nicholas Senn, J. Collins Warren, L. A. Stinson, P. S. Connor, and the Editors. F. W. M.

---

THE THREE ETHICAL CODES. That of the American Medical Association; its Constitution, By-Laws, Amendments, etc. That of the American Institute of Homœopathy and that of the National Eclectic Medical Society. Limp cloth, round corners, 55 pages, postpaid 50 cents. The Illustrated Medical Journal Company, Publishers, Detroit, Mich.

By comparing the Code of the Homœopathic Society with that of the American Medical Association, it will be found that several sections of the former are similar to parts of the latter's code. The Eclectic Code is worthy of mention for its brevity.

It will repay every physician to get this little book and inform himself on the articles of faith of these three so-called schools of medicine.

---

READERS desiring to have special reprints of the Jenner Centenary Number or of G. Kirtland's colored illustrations of cowpox and inoculated small-pox separately, are requested to make early application to the general secretary, as it will be impossible to keep the type standing or to preserve the lithographic plates much longer.—*Brit. Med. Jour., June 6.*

